# Integrating Super-Resolution with Deep Learning for Enhanced Periodontal Bone Loss Segmentation in Panoramic Radiographs

**DOI:** 10.3390/bioengineering11111130

**Published:** 2024-11-08

**Authors:** Vungsovanreach Kong, Eun Young Lee, Kyung Ah Kim, Ho Sun Shon

**Affiliations:** 1 Department of Big Data, Chungbuk National University, Cheongju 28644, Republic of Korea; kv.sovanreach@gmail.com; 2 Department of Oral and Maxillofacial Surgery, Chungbuk National University Hospital, Cheongju 28644, Republic of Korea; ley926@chungbuk.ac.kr; 3 Department of Oral and Maxillofacial Surgery, College of Medicine, Chungbuk National University, Cheongju 28644, Republic of Korea; 4 Department of Biomedical Engineering, College of Medicine, Chungbuk National University, Cheongju 28644, Republic of Korea; 5 Biomedical Research Institute, Chungbuk National University Hospital, Cheongju 28644, Republic of Korea; 6 Medical Research Institute, School of Medicine, Chungbuk National University, Cheongju 28644, Republic of Korea

**Keywords:** periodontal bone loss, semantic segmentation, super resolution, deep learning, SRGAN, U-Net

## Abstract

Periodontal disease is a widespread global health concern that necessitates an accurate diagnosis for effective treatment. Traditional diagnostic methods based on panoramic radiographs are often limited by subjective evaluation and low-resolution imaging, leading to suboptimal precision. This study presents an approach that integrates Super-Resolution Generative Adversarial Networks (SRGANs) with deep learning-based segmentation models to enhance the segmentation of periodontal bone loss (PBL) areas on panoramic radiographs. By transforming low-resolution images into high-resolution versions, the proposed method reveals critical anatomical details that are essential for precise diagnostics. The effectiveness of this approach was validated using datasets from the Chungbuk National University Hospital and the Kaggle data portal, demonstrating significant improvements in both image resolution and segmentation accuracy. The SRGAN model, evaluated using the Peak Signal-to-Noise Ratio (PSNR) and Structural Similarity Index Measure (SSIM) metrics, achieved a PSNR of 30.10 dB and an SSIM of 0.878, indicating high fidelity in image reconstruction. When applied to semantic segmentation using a U-Net architecture, the enhanced images resulted in a dice similarity coefficient (DSC) of 0.91 and an intersection over union (IoU) of 84.9%, compared with 0.72 DSC and 65.4% IoU for native low-resolution images. These results underscore the potential of SRGAN-enhanced imaging to improve PBL area segmentation and suggest broader applications in medical imaging, where enhanced image clarity is crucial for diagnostic accuracy. This study also highlights the importance of further research to expand the dataset diversity and incorporate clinical validation to fully realize the benefits of super-resolution techniques in medical diagnostics.

## 1. Introduction

Periodontal disease ranks among the most prevalent chronic conditions globally, with its incidence in South Korea predicted to increase by 5.9% between 2018 and 2022 [1]. This increase positions periodontal disease as the top outpatient diagnosis, imposing a substantial burden on national healthcare expenditures. Effective prevention and management can significantly mitigate the incidence of periodontal disease. However, many patients delay seeking treatment until the symptoms become severe, complicating early diagnostic efforts. This disease is associated with a range of complications for patients, including pain, inflammation, gum recession, and an increased risk of tooth loss. These issues can significantly affect oral function, esthetics, and overall quality of life. Moreover, untreated periodontal disease has been linked to broader systemic health concerns, highlighting the importance of accurate early diagnosis and effective management to prevent progression.

Current diagnostic methods for early detection and identification of periodontal disease include clinical assessments, such as probing depth measurements and clinical attachment level evaluation, along with radiographic imaging through panoramic and periapical radiographs. These diagnostic methods are highly reliant on the clinician’s assessment of the patient’s dental condition and analysis of the patient’s panoramic radiographs. Generally, it is limited by subjective interpretation, reliance on clinician expertise, and, often, suboptimal image resolution in radiographic assessments. These limitations can result in inconsistent early detection and variable assessments of disease severity. Deep learning techniques address these challenges by offering automated, high-precision analysis that enhances image resolution and segmentation accuracy, thereby supporting more objective and reliable diagnoses.

This study introduces an approach that integrates super-resolution and deep learning techniques to reconstruct high-resolution images and enhance the PBL segmentation model. Specifically, this approach implements generative adversarial networks (GANs) for super-resolution, transforming low-resolution images into high-resolution versions that reveal finer structural details within the images. GANs are capable of generating realistic, detailed images, making them well-suited for super-resolution tasks where high image clarity is required. This study aims to introduce an approach that integrates super-resolution and deep learning techniques to reconstruct high-resolution images, thereby enhancing the segmentation model’s accuracy in identifying periodontal bone loss (PBL) areas. This capability is crucial for addressing the limitations of panoramic radiographs, which are commonly used to assess the critical indicators of periodontal health.

Deep learning-based segmentation models were then trained to achieve precise segmentation of the PBL area within the panoramic radiograph. This integration not only surpasses the existing limitations associated with imaging resolution but also facilitates more accurate and dependable diagnoses of periodontal diseases. Consequently, this methodology significantly supports dental professionals in developing effective treatment plans based on precise diagnostic information, thereby potentially improving patient care outcomes.

This research underwent experimental validation using data sourced from the Chungbuk National University Hospital (CBNUH) and the Kaggle data portal. This paper outlines a methodology that merges super-resolution and deep learning techniques to optimize the segmentation of PBL in panoramic radiographs. This study aimed to enhance the initial diagnosis and accurate evaluation of periodontal diseases with the ultimate goal of improving patient treatment results.

This study introduces an approach by integrating SRGAN with U-Net-based semantic segmentation to enhance PBL segmentation in panoramic radiographs. Unlike traditional super-resolution methods, SRGAN effectively reconstructs fine anatomical details, which are critical for accurate diagnostic assessment. The research contributes to the literature by demonstrating how super-resolution can directly improve segmentation performance, leading to more precise diagnostics. This methodology not only supports advancements in dental radiographic imaging but also suggests potential applications in other areas of medical imaging where image clarity and segmentation accuracy are essential for reliable clinical outcomes.

The remainder of this article is organized as follows: Section 2 provides a review of recent advancements in super-resolution and deep learning applications in medical imaging, specifically focusing on segmentation techniques. Section 3 details the methodology, including dataset preparation, SRGAN architecture, and U-Net model for segmentation. Section 4 presents the experimental results with evaluation metrics used to assess model performance. Section 5 discusses the results, comparing SRGAN-enhanced segmentation outcomes to baseline methods. Finally, Section 6 concludes the study by summarizing key findings, implications, and directions for future research.

## 2. Related Works

Recent advances in artificial intelligence (AI) have led to the development of various products and services, particularly image processing and recognition. Advances in computer technology have significantly enhanced image recognition capabilities within computer vision. This process involves analyzing and interpreting the meanings embedded within image data using AI algorithms [2].

Deep Neural Networks have demonstrated outstanding performances in the fields of image processing and computer vision. These networks utilize deep learning-based models to extract and process features from images. Deep learning autonomously extracts data features during the learning process, providing a consistent feature extraction model, regardless of the imaging device or specific area. One such deep learning model that is frequently used for object segmentation is UNet, which improves upon the limitations of traditional Convolutional Neural Networks (CNNs) by utilizing a fully convolutional network structure. This consists of an encoder and decoder setup, yielding excellent results in semantic segmentation [3,4].

Deep learning offers distinct advantages over traditional diagnostic methods and standard machine learning techniques, particularly in medical image analysis. Unlike traditional methods that depend on manual feature selection, deep learning models automatically learn complex patterns directly from the data, leading to greater accuracy and objectivity. Additionally, deep learning models are capable of processing large datasets and capturing subtle variations, providing a level of scalability and precision that is difficult to achieve with conventional approaches.

Significant progress has also been made in the application of deep learning algorithms for the analysis of panoramic dental images. Shon et al. (2022) proposed a deep learning framework developed for classifying various stages of periodontitis that uses patient-specific panoramic radiographic data to detect bone loss and periapical boundaries, and categorizes periodontitis based on radiographic bone-level criteria. This framework has proven to be highly accurate, making it a valuable tool for dental professionals in diagnosing and planning treatment [5].

Moreover, new methods that combine deep learning and image-processing technologies have been developed by Li et al. (2023) to detect teeth and classify dental issues in panoramic X-ray images. These approaches include segmentation and classification models, which are essential for accurately diagnosing patterns of PBL and bifurcation defects [6].

Research employing deep learning models, such as Faster R-CNN, proposed by Ryu et al. (2023), has been conducted to detect teeth affected by periodontal damage on panoramic radiographs. These models have contributed to enhancing the consistency and reproducibility among examiners, thereby increasing the reliability of periodontal assessments and demonstrating their potential for automating the diagnosis of periodontitis [7].

Advanced deep learning architectures, such as ResNet and attention mechanisms, have been introduced to more effectively identify dental diseases in radiographic images. These technological advancements emphasize critical areas within images, aiding in the detection of subtle anomalies that may be overlooked by conventional methods [8].

Standard GANs focus on generating realistic images by using an adversarial network structure, but they are not specifically optimized for high-resolution reconstruction. SRGAN (Super-Resolution GAN), in contrast, is designed to create high-resolution images from low-resolution inputs by using a perceptual loss function that emphasizes structural accuracy. This feature makes SRGAN particularly suitable for medical imaging tasks where precise anatomical detail is essential. The application of SRGAN in this study aims to enhance the accuracy of periodontal bone loss segmentation by reconstructing high-fidelity images that support a more accurate analysis and diagnosis.

Research on Super-Resolution Generative Adversarial Networks (SRGANs) has primarily focused on enhancing image resolution with potential applications in dental imaging, including images used for diagnosing periodontal diseases. Although no specific studies have directly applied SRGAN to the treatment or diagnosis of periodontal disease, research utilizing this technology has yielded positive results [9].

SRGAN has significantly advanced the field of image super-resolution by transforming low-resolution images into high-resolution images. Originally proposed by Ledig et al. (2017), SRGAN utilizes a generative adversarial network to create more realistic and detailed high-resolution images, overcoming the limitations of traditional methods that primarily focus on minimizing the Mean Squared Error (MSE) in the image restoration process. This dual-network approach enables the creation of more realistic high-resolution images [10].

Previous studies in medical imaging indicate that segmentation accuracy significantly benefits from enhanced image resolution, as it allows models to capture finer anatomical details. In periodontal disease diagnostics, accurate segmentation of periodontal bone loss (PBL) is essential for early detection and effective treatment planning. This study builds on these insights by integrating SRGAN with U-Net for PBL segmentation, a novel approach aimed at addressing the limitations of traditional radiographic methods.

## 3. Materials and Methods

**Figure 1:**
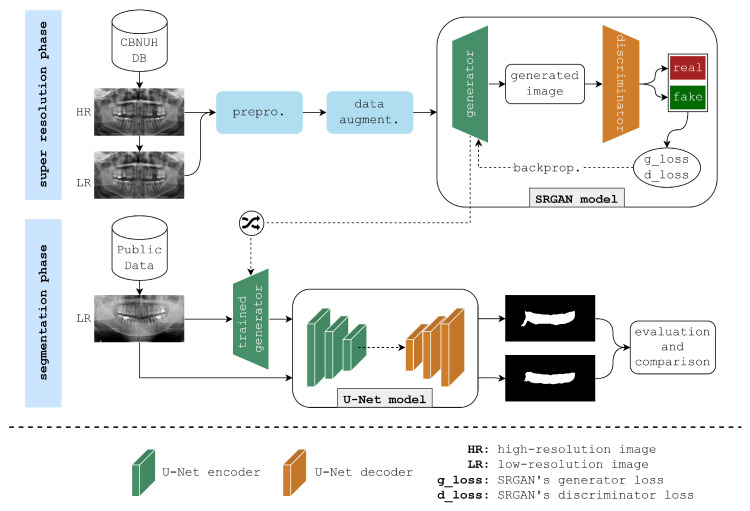
The architecture of the proposed framework for enhancing periodontal bone loss (PBL) segmentation in panoramic radiographs.

This paper presents the framework illustrated in Figure 1 to enhance the semantic segmentation of PBL areas in panoramic radiographs. The framework begins with two datasets of panoramic radiographs: a high-quality CBNUH dataset and an open-source dataset from Kaggle. First, the CBNUH dataset underwent preprocessing, which includes rescaling images to a consistent resolution, followed by normalization to ensure uniform pixel values. This dataset was then split into low-resolution and high-resolution versions to serve as the training input for the SRGAN model. To increase the robustness of the model given the limited sample size, data augmentation techniques, horizontal flips, ±15-degree rotations, and image shifts, were applied to the CBNUH images. Next, datasets from CBNUH were used to train the SRGAN model, which learned to reconstruct high-resolution images. After training, the SRGAN model was tested on low-resolution images from the Kaggle dataset, which were enhanced to high-resolution versions. This testing phase allowed us to compare the results of the SRGAN enhancement on previously unseen low-resolution data, highlighting its generalizability and effectiveness for segmentation applications. The enhanced high-resolution images received from SRGAN were then input into the U-Net model for segmentation, which applied an encoder–decoder structure with skip connections to accurately isolate PBL regions. The final output from this step was a segmentation mask that identified PBL areas with enhanced precision, supporting more accurate diagnostic assessments. This section provides a detailed explanation of the framework, beginning with data collection and processing, and concluding with an evaluation of the trained models.

### 3.1. Panoramic Radiograph Datasets

**Figure 2:**
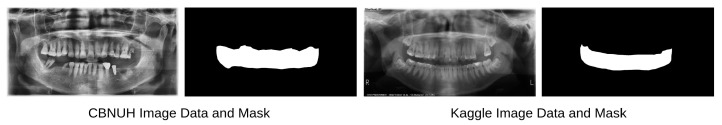
Samples of panoramic radiographs and PBL masks from each data source.

Panoramic radiographs from two different data sources were used in this experiment: the CBNUH dataset and an open-source dataset publicly available in Kaggle [11]. These data were carefully reviewed and manually labeled by professional dentists to ensure that annotated masks were correctly and accurately labeled. The CBNUH dataset was approved by the Institutional Review Board of the CBNUH (CBNUH 2021-11-011) before the experiment began. CBNUH data were collected from 100 patients who underwent upper- and lower-jaw scans during their hospital visits. In the proposed framework, the CBNUH and public datasets were used to train the super-resolution and semantic segmentation models, respectively. Figure 2 shows a random sample image from each data source with its corresponding manually labeled PBL mask.

Table 1 lists the number of X-ray images and masks for each dataset used in this experiment. Owing to the nature and small size of the sample, data preprocessing and augmentation, which will be explained in the next section, were applied to prevent overfitting and increase the accuracy of both super-resolution and semantic segmentation.

### 3.2. Data Preprocessing and Augmentation

Several data preprocessing techniques were implemented to prepare panoramic radiograph images for both semantic segmentation and super-resolution models [12]. First, image normalization was applied to scale the pixel values between 0 and 1, primarily to enhance model generalization. Second, for the super-resolution model, the images were downscaled, resulting in the creation of two versions of the CBNUH dataset: low- and high-resolution. The low-resolution version is important for training the super-resolution model, which is supposed to accurately upscale low-resolution images to high resolution. Using these two versions, the super-resolution model can learn image patterns and accurately reconstruct high-resolution versions from low-resolution input images.

**Figure 3:**
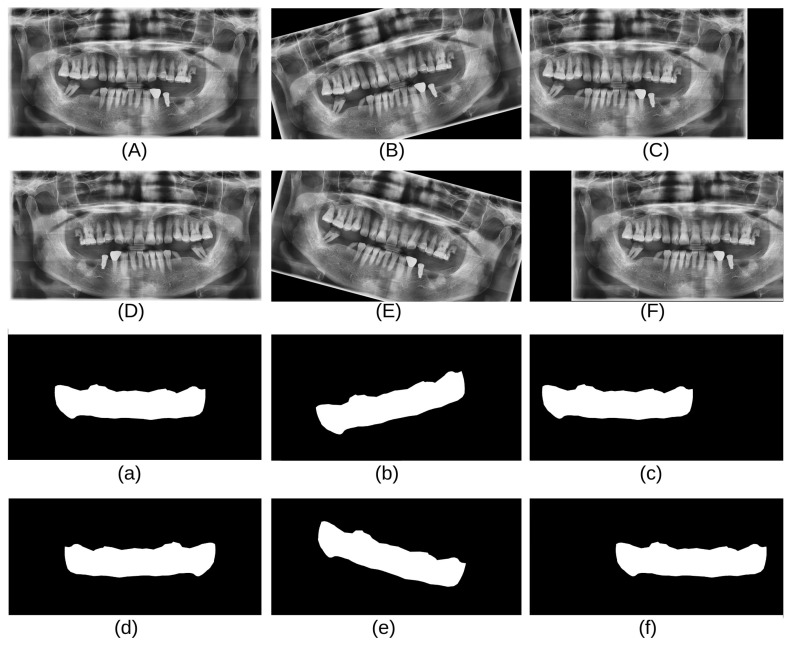
Images and masks after data augmentation techniques were applied: (**A**) original, (**B**) 15-degree rotation, (**C**) left shift, (**D**) horizontal flip, (**E**) −15-degree rotation, and (**F**) right shift. Each mask (**a**–**f**) aligns with its respective augmented image (**A**–**F**).

As shown in Table 1, the CBNUH dataset has a limited number of images, which can result in a low model accuracy. To address this, several data augmentation techniques, such as horizontal flips, rotations (15 degrees, −15 degrees), and shifting, were applied to increase the size of the dataset. These techniques help improve the orientation robustness of the model and prevent overfitting [13]. In this study, data augmentation techniques were applied to the CBNUH dataset, resulting in a fivefold increase in the original image count. Consequently, the dataset expanded to a total of 600 images, comprising both the augmented and original images. Figure 3 shows examples of the data obtained after applying each augmentation technique.

### 3.3. Super-Resolution Generative Adversarial Network (SRGAN)

#### 3.3.1. SRGAN Overview and Network Architecture

The super-resolution algorithm was specifically used to enhance the quality of panoramic radiographs, which also significantly improved the precision of PBL segmentation. SRGAN is a deep learning technique developed for single-image super-resolution that can improve image quality using a generator–discriminator architecture. SRGAN is trained in an adversarial manner, which is responsible for creating a realistic high-resolution image, whereas the discriminator attempts to distinguish between a real high-resolution image and an image generated by the generator [14]. In this framework, SRGAN was implemented as a super-resolution technique due to its superior ability to reconstruct high-resolution images with preserved structural details, which is crucial for the accurate segmentation of periodontal bone loss (PBL) areas [10]. Unlike simpler techniques, such as contrast enhancement or traditional super-resolution methods, SRGAN utilizes a generator–discriminator network to learn complex image features, thus retaining essential anatomical structures while minimizing noise. The resulting loss value was used for backpropagation in the other training epochs. Through this process, the generator component of the trained SRGAN model learned to create highly realistic high-resolution images from low-resolution input images.

SRGAN consists of two main components, which are the generator and discriminator networks. The generator network converts low-resolution images into high-resolution images using multiple residual blocks (ResNet) to capture complex features [8,15]. Each residual block consists of convolutional layers, batch normalization, and an activation function (ReLU) [16]. After a series of residual blocks, an upsampling layer is added to increase the spatial resolution of the feature maps and produce a high-resolution output. On the other hand, the discriminator is designed to differentiate between actual high-resolution images and the images generated by the generator. It employs multiple convolutional layers with progressively increasing filter sizes, followed by batch normalization and an activation function (LeakyReLU) [17]. These layers enable the discriminator to carefully and accurately distinguish between real and generated images. The SRGAN framework was trained using two key loss functions: adversarial and content losses. Adversarial loss drives the generator to produce outputs that can fool the discriminator, whereas content loss ensures that the generated images preserve essential content and texture details. Content loss is often calculated using the MSE or feature-based loss from a pretrained network, such as a Visual Geometry Group [18].

#### 3.3.2. Training Setup

The SRGAN model was trained using panoramic radiographs as input, with the aim of generating high-resolution images that could be used to improve the performance of the subsequent U-Net model for semantic segmentation of PBL. The dataset was split into 70% (420 images) training and 30% (180 images) test sets, with the training set used to train the model and the test set to evaluate it. The training process involved the use of a learning rate of $1\times 10^{-4}$, $1\times 10^{-5}$, $1\times 10^{-6}$ for both the generator and discriminator, with an Adam optimizer configured with $\beta_{1}$ = {0.5, 0.7, 0.9} and $\beta_{2}$ = {0.997, 0.998, 0.999}. SRGAN was trained for 250 epochs with a batch size of 16, which was determined to provide a good balance between training stability and speed. The model was trained using a combination of perceptual loss, adversarial loss, and pixel-wise MSE with weights of 1.0, 0.05, and 0.01, respectively, to ensure high-fidelity and visually appealing reconstructions.

### 3.4. Semantic Segmentation with U-Net

#### 3.4.1. U-Net Overview and Network Architecture

The U-Net model was originally developed for semantic segmentation of medical images because of its ability to extract fine-grained details from medical images [4]. It is a fully convolutional network specifically designed to work with limited annotated data and is well suited for semantic segmentation tasks, such as detecting PBL in panoramic radiographs. In this framework, only the Kaggle dataset was used for U-Net segmentation. The CBNUH images were specifically employed to train the SRGAN model to enhance image resolution, while the U-Net segmentation model was trained and evaluated using the original and SRGAN-enhanced versions of the Kaggle dataset. This allowed for a consistent comparison of segmentation performance with and without super-resolution enhancement. By comparing the performances of the models trained on these datasets, this study aimed to assess how super-resolution enhances the accuracy and precision of the segmentation outcomes.

U-Net was designed as an encoder–decoder network [19]. The encoder is responsible for compressing and extracting the features in an image. This is achieved through a series of convolutional layers, ReLU activation, and max-pooling steps, which gradually shrink the size of the image, while increasing the depth of its feature maps. This allowed the network to capture both nitty-gritty details and big-picture concepts at different image levels.

On the opposite side, the decoder rebuilds the image resolution. It uses upsampling techniques, often through transposed convolutions, to slowly increase the spatial dimensions of feature maps. A novel trick in the U-Net sleeve is the use of skip connections, which link matching layers from the encoder to the decoder. These skip connections allow the model to blend high-resolution features from the squeezing path with the upsampled features, ensuring that no fine details are lost in the shuffle during segmentation.

**Figure 4:**
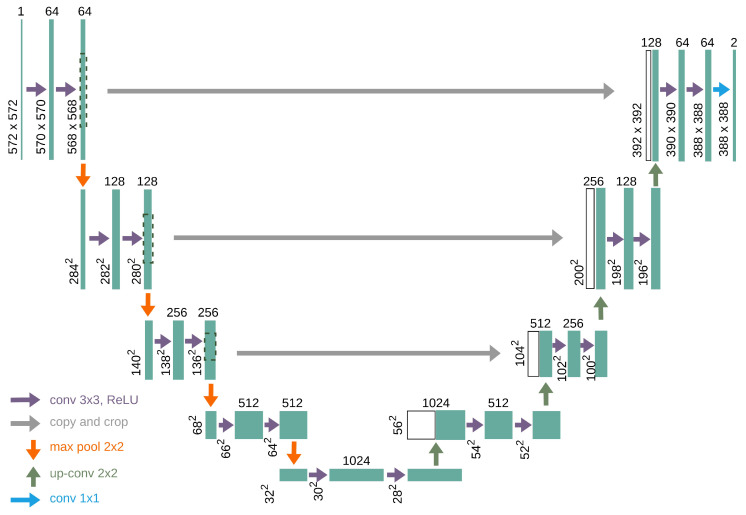
U-Net architecture overview, highlighting both encoder and decoder components [4].

The final output layer of the U-Net model is a 1 × 1 convolution that maps the feature maps to the desired number of classes, which, in this case, was two (0 and 1), followed by an activation function to produce the segmentation mask. In the context of PBL segmentation, this architecture effectively balances the need for detailed local information and broader contextual understanding, making it highly effective for medical image analysis. Figure 4 shows the detailed architecture of the U-Net model used in this framework.

Semantic segmentation of periodontal bone loss (PBL) areas is a crucial process that enables accurate identification and delineation of bone loss regions. This precision is essential for determining the severity of periodontal disease, monitoring its progression, and evaluating treatment efficacy. By automating segmentation, the model reduces reliance on manual analysis, which can be subjective and inconsistent, thus promoting diagnostic consistency and supporting more reliable clinical decision-making.

#### 3.4.2. Training Setup

Following super-resolution enhancement, the U-Net model was implemented for semantic segmentation of the PBL in enhanced panoramic radiographs. The Kaggle dataset was split into 70% (419 images) training and 30% (179 images) test sets to train and evaluate the U-Net model, respectively. The U-Net was initialized with a learning rate of $1\times 10^{-3}$, $1\times 10^{-4}$, $1\times 10^{-5}$, using the Adam optimizer with the same $\beta_{1}$ and $\beta_{2}$ values as SRGAN. The model was trained for 500 epochs with a batch size of 16 owing to the increased resolution and complexity of the images. The cross-entropy loss function was used, coupled with a dice coefficient, as a metric to evaluate the segmentation performance.

### 3.5. Experimental Environment Setup

The experimental setup for this study was designed to handle the computational demands of training both the SRGAN and U-Net models for semantic segmentation. The experiments were conducted on a dedicated server equipped with two NVIDIA GTX 1080 Ti GPUs with 12 GB of VRAM, allowing efficient processing of large image datasets. The server was powered by an Intel Xeon Gold 6226R CPU with 32 cores and 64 GB of DDR4 RAM running on Ubuntu 24.04 LTS. This setup provides the necessary computational power and memory to handle the extensive training procedures required for both models.

### 3.6. Evaluation Metrics

In this study, the SRGAN and U-Net models were evaluated using a set of quantitative metrics designed to assess both the quality of super-resolved images and the performance of the semantic segmentation of PBL. The metrics were selected to ensure a comprehensive evaluation of the effectiveness of the models in enhancing image resolution and accurately segmenting the regions of interest.

#### 3.6.1. SRGAN Model Evaluation

The performance of SRGAN was primarily evaluated using two key metrics: Peak Signal-to-Noise Ratio (PSNR) and Structural Similarity Index Measure (SSIM):

- PSNR: PSNR is a widely used metric for assessing the quality of reconstructed images in terms of fidelity to the original high-resolution images [20]. It is defined as
  (1) $$\mathrm{PSNR}=10\cdot \log_{10}\left(\frac{MAX_{I}^{2}}{\mathrm{MSE}}\right)$$
  where $MAX_{I}$ is the maximum possible pixel value of the image and MSE is the Mean Squared Error between the super-resolved image and the ground truth high-resolution image. A higher PSNR indicates better image quality with reduced noise and distortions.
- SSIM: SSIM is a perceptual metric that quantifies the similarity between two images based on structural information [21]. It considers luminance, contrast, and structure comparison, making it more aligned with human visual perception than the PSNR. SSIM is defined as
  (2) $$\mathrm{SSIM}\left(x,y\right)=\frac{\left(2\mu_{x}\mu_{y}+C_{1}\right)\left(2\sigma_{xy}+C_{2}\right)}{\left(\mu_{x}^{2}+\mu_{y}^{2}+C_{1}\right)\left(\sigma_{x}^{2}+\sigma_{y}^{2}+C_{2}\right)}$$
  where *x* and *y* are the original and super-resolved images, $\mu_{x}$ and $\mu_{y}$ are the mean intensities, $\sigma_{x}$ and $\sigma_{y}$ are the standard deviations, and $\sigma_{xy}$ is the covariance of *x* and *y*. Constants $C_{1}$ and $C_{2}$ are used to stabilize the division. SSIM values range from −1 to 1, with values closer to 1 indicating greater structural similarity.

#### 3.6.2. U-Net Model Evaluation

The performance of the U-Net model in segmenting the PBL was evaluated using the dice similarity coefficient (DSC) and intersection over union (IoU):

- DSC: The dice coefficient, also known as F1 score, is a statistic used to gauge the similarity between two sets [22]. In the context of image segmentation, it measures the overlap between the predicted segmentation map and the ground truth, defined as
  (3) $$\mathrm{DSC}=\frac{2\cdot |A\cap B|}{\left|A|+|B\right|}$$
  where *A* is the set of pixels corresponding to the ground truth and B is the set of pixels predicted by the model. A DSC closer to 1 indicates a higher degree of overlap and, thus, better segmentation performance.
- IoU, also known as the Jaccard Index, is another metric used to evaluate segmentation accuracy [23]. It is defined as
  (4) $$\mathrm{IoU}=\frac{\left|A\cap B\right|}{\left|A\cup B\right|}$$
  Similar to the DSC, a higher IoU value indicates a better match between the predicted segmentation and ground truth. The IoU is particularly useful for understanding a model’s performance across different classes and is often used in conjunction with the DSC for a more robust evaluation.

## 4. Results

This section presents the results obtained from the application of the SRGAN model to enhance the resolution of panoramic radiographs and the subsequent performance improvement in semantic segmentation for PBL detection. The analysis is divided into three subsections focusing on the performance of the super-resolution model, semantic segmentation model, and the impact of super-resolution on segmentation accuracy and precision.

### 4.1. Super Resolution Model Performance

**Figure 5:**
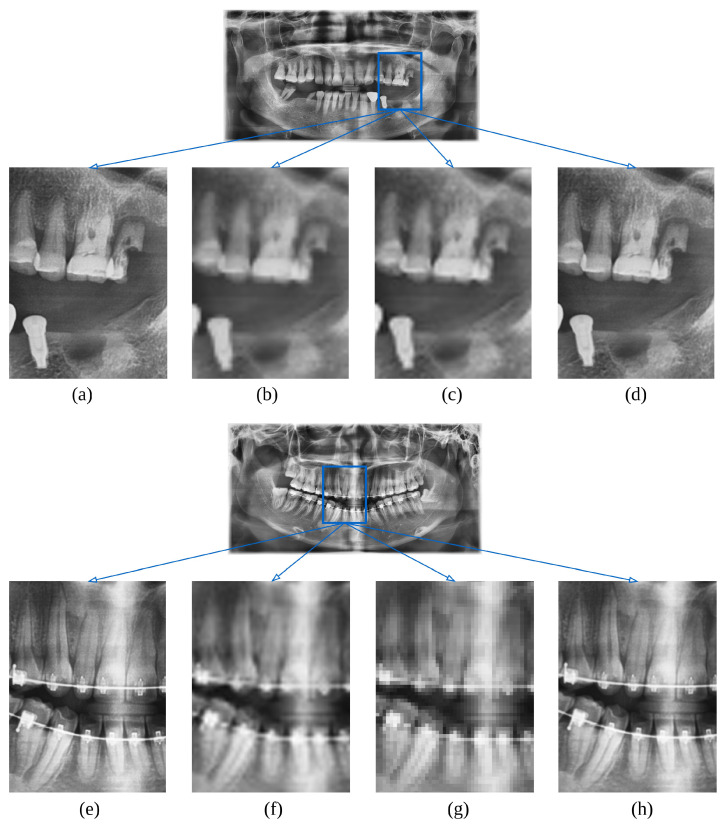
Comparison of image resolution enhancement techniques: (**a**,**e**) high-resolution original, (**b**,**f**) low-resolution original, (**c**,**g**) bicubic interpolation, and (**d**,**h**) SRGAN enhancement.

The performance of the SRGAN model was evaluated using the PSNR and SSIM as metrics to assess the quality of image enhancement. The model was trained and tested using a dataset of 600 original and augmented low-resolution images and their corresponding high-resolution images. After training, the SRGAN model achieved a PSNR of 30.10 dB and an SSIM of 0.878, indicating a high degree of fidelity in the image reconstruction. Figure 5 presents a visual comparison of the original high-resolution, original low-resolution, bicubic interpolation, and enhanced images. SRGAN-enhanced images displayed superior anatomical clarity and preserved fine structural details, enabling more precise segmentation of periodontal bone loss (PBL) areas compared to both low-resolution and bicubic-interpolated images. Quantitatively, SRGAN enhancement achieved higher PSNR and SSIM values, reflecting improved image fidelity and segmentation performance. The SRGAN images exhibited significantly improved clarity and detail, which are essential for accurate subsequent analyses.

Table 2 summarizes the quantitative performance of the SRGAN model in terms of the PSNR and SSIM. Among the tested configurations, the model with 32 residual blocks, a learning rate of $1\times 10^{-6}$, and beta values of (0.5, 0.999) achieves the highest PSNR (30.10 dB) and SSIM (0.878). These results indicate that this configuration provides the most effective balance in reconstructing high-fidelity, structurally accurate images. This finding highlights the importance of deeper network architectures and lower learning rates in achieving superior resolution enhancement for panoramic radiographs.

### 4.2. Semantic Segmentation Model Performance

**Figure 6:**
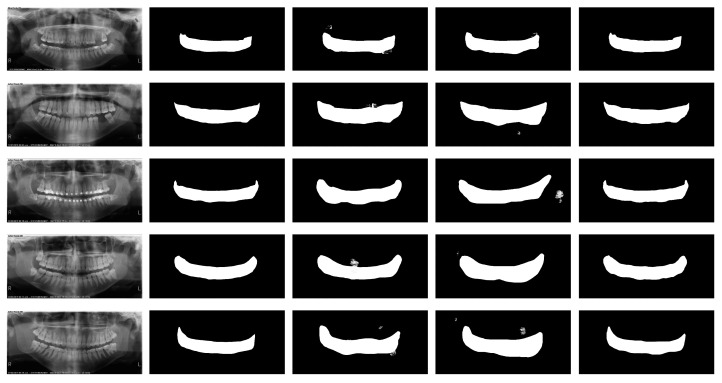
Visual comparison of semantic segmentation on native, bicubic-interpolated, and enhanced images.

The semantic segmentation model, based on the U-Net architecture, was assessed on both the original low-resolution images and enhanced images. The model performance was quantified using DSC and IoU metrics. On native low-resolution images, the segmentation model achieved a DSC of 0.72 and an IoU of 65.4%. An improvement of 0.91 in DSC and 84.9% in IoU was achieved when the enhanced images were used as the input data. Table 3 summarizes the performance metrics of the segmentation model across the different resolution modalities. Figure 6 illustrates the comparative segmentation results for the native, bicubic interpolation, and enhanced images, highlighting the detailed recovery of the PBL area contours in the enhanced images. This improvement is quantitatively supported by the improved metrics listed in Table 3.

## 5. Discussion

The results obtained in this study demonstrate the efficacy of using SRGAN to enhance the resolution of panoramic radiographs, which, in turn, significantly improves the precision and accuracy of semantic segmentation models for detecting PBL. The application of SRGAN for image super-resolution led to a notable improvement in segmentation accuracy, as demonstrated by the increased IoU (84.9%) and DSC values (0.91) in SRGAN-enhanced images. Enhanced images facilitated better delineation of fine PBL structures that were challenging to discern in lower-resolution images, underscoring the potential of super-resolution techniques to improve segmentation outcomes in medical imaging. This section discusses the implications of these findings, limitations of the current study, and potential areas for future research.

### 5.1. Impact of Super-Resolution on Segmentation

The application of SRGAN for image super-resolution significantly affects the accuracy and precision of semantic segmentation. The enhanced images showed a noticeable improvement in the semantic segmentation ability to delineate finer structures related to the PBL, which were otherwise obscured in lower-resolution images.

Table 4 provides a statistical analysis of the improvement in segmentation performance by comparing native and enhanced images across multiple samples. The *p*-values obtained from the paired *t*-test confirmed the statistical significance of these improvements.

Both qualitative and quantitative findings underscore the impact of the SRGAN model on enhancing panoramic radiographs for periodontal bone loss (PBL) segmentation. Qualitatively, SRGAN-enhanced images revealed finer anatomical details, allowing more precise delineation of PBL structures, particularly in subtle regions that were less visible in lower-resolution images. Quantitatively, the SRGAN model achieved a PSNR of 30.10 dB and an SSIM of 0.878, indicating high reconstruction fidelity and structural accuracy. These improvements in image quality translated to better segmentation results, as the U-Net model achieved a DSC of 0.91 and IoU of 84.9% on SRGAN-enhanced images, compared to a DSC of 0.72 and IoU of 65.4% on native images. These findings highlight SRGAN’s effectiveness in enhancing image quality for improved segmentation accuracy, supporting its potential in medical imaging applications where high-resolution detail is critical. The results indicate that using SRGAN not only enhances image quality but also significantly improves the performance of semantic segmentation models in detecting and analyzing PBL in panoramic radiographs.

### 5.2. Implication of Findings

The improvement in image quality, as evidenced by higher PSNR and SSIM scores in the SRGAN-enhanced images, has direct implications for clinical diagnostics. Enhanced image resolution allows better visualization of subtle bone structures, which is crucial for early diagnosis and treatment planning in periodontology. The increase in the DSC and IoU metrics when using enhanced images indicates more precise segmentation, potentially leading to more accurate assessments of bone loss severity and progression.

Furthermore, the application of SRGAN could be extended beyond panoramic radiographs to other types of medical imaging where detailed recovery is crucial, such as in mammography, angiography, or even non-medical fields requiring detailed image analysis.

### 5.3. Limitations

Although these findings are promising, this study has several limitations. First, training of the SRGAN model was limited to a dataset of 100 unique images. A larger dataset may help generalize the model’s performance across more varied cases. Second, this study focused exclusively on the quantitative metrics of image and segmentation quality. Clinical validation through expert radiologist assessment is necessary to corroborate the clinical utility of enhanced images. In addition, the computational demands of SRGAN models are significant, potentially limiting their use in real-time applications without specialized hardware.

### 5.4. Future Research

Future research can address these limitations by expanding the size and diversity of the dataset to train more robust SRGAN models. It would also be beneficial to include clinical evaluations as a part of the study to directly link image quality improvements to enhanced diagnostic outcomes.

Exploring the integration of SRGAN-enhanced imaging with real-time diagnostic systems could also be a valuable area of research, particularly with the development of more efficient GAN architectures that reduce computational overhead. Moreover, investigating the application of super-resolution techniques, similar to other imaging modalities, could broaden the impact of this technology in medical diagnostics.

Finally, further research might explore combining SRGAN with other forms of AI, such as deep learning models, which can directly predict clinical outcomes from enhanced images, thereby creating an end-to-end diagnostic tool that could revolutionize the field of radiographic imaging in dentistry and beyond.

## 6. Conclusions

This study investigated the efficacy of the SRGAN technique for enhancing the resolution of panoramic radiographs and its impact on the precision and accuracy of semantic segmentation for PBL detection. The results demonstrate that SRGAN significantly improves both image quality (as measured by PSNR and SSIM) and the effectiveness of semantic segmentation models (evidenced by higher DSC and IoU values).

The implementation of SRGAN led to a substantial enhancement in image detail, which facilitated a more accurate delineation of the PBL. This improvement is crucial for the early detection and accurate assessment of periodontal diseases, which is key to effective treatment planning. The potential of super-resolution techniques extends beyond dental radiography to broader medical imaging applications, where enhanced image clarity can substantially affect diagnostic capabilities. Despite its promising results, this study acknowledges limitations, such as the restricted size of the training dataset and the absence of clinical validation. Addressing these limitations in future studies could clarify the role of super-resolution techniques in medical imaging.

In the future, it is recommended that subsequent studies expand the dataset to train more generalized SRGAN models, integrate clinical evaluation into the validation process, and explore the application of super-resolution in real-time diagnostic systems. Such advancements could pave the way for SRGANs not only to improve diagnostic imaging quality but also to enhance clinical outcomes across various fields of medicine.

In conclusion, the integration of SRGAN into the preprocessing of medical images represents a significant advancement in computational imaging with profound implications for clinical practice. The ability to effectively improve image resolution and utilize enhanced images for precise medical analysis promises to revolutionize medical diagnostics and treatment strategies in periodontology.

## Figures and Tables

**Table 1:** Overview of dataset characteristics, including the number of images and masks, and image size from each data source.

Dataset	Number of X-Ray Images	Number of Masks	Image Size (Pixels)	IRB Approval ^1^
CBNUH	100	100	2652 × 1372	YES
Kaggle (Public)	598	598	2000 × 942	NO

^1^ IRB: Institutional Review Board.

**Table 2:** Quantitative performance of SRGAN on panoramic radiographs: PSNR and SSIM metrics.

Hyperparameters	PSNR	SSIM
Residual blocks: 16, lr: $1\times 10^{-5}$, betas: (0.5, 0.999)	29.45	0.865
Residual blocks: 8, lr: $1\times 10^{-4}$, betas: (0.9, 0.999)	28.30	0.852
Residual blocks: 32, lr: $1\times 10^{-6}$, betas: (0.5, 0.999)	30.10	0.878

**Table 3:** Performance metrics of semantic segmentation across different image resolutions.

Image Type	DSC	IoU	Accuracy	Sensitivity	Specificity
Low resolution (native)	0.72	65.4%	80.2%	78.5%	82.1%
Bicubic interpolation	0.76	68.7%	83.5%	81.3%	85.0%
SRGAN-enhanced	0.91	84.9%	90.4%	88.7%	92.0%

**Table 4:** Statistical analysis of segmentation performance improvement with SRGAN enhancement.

Metric	Mean Improvement	Standard Deviation	*p*-Value
DSC	0.08	0.015	0.001
IoU	6.2%	1.8%	0.003

## Data Availability

Dataset available on request from the authors.

## Abbreviations

The following abbreviations are used in this manuscript:

PBL	Periodontal Bone Loss
SRGANs	Super-Resolution Generative Adversarial Networks
GANs	Generative adversarial networks
PSNR	Peak Signal-to-Noise Ratio
SSIM	Structural Similarity Index Measure
DSC	Dice similarity coefficient
IoU	Intersection over union
